# Healthcare workers’ and managers’ uses of mobile phone messaging applications (apps) in their daily work in South Africa: a survey

**DOI:** 10.1093/oodh/oqag005

**Published:** 2026-02-17

**Authors:** Simon Lewin, Janan Janine Dietrich, Mamakiri Mulaudzi, Tondani Mudau, Lerato Tsotetsi, Tanya Doherty

**Affiliations:** Department of Health Sciences Ålesund, Faculty of Medicine and Health Sciences, Norwegian University of Science and Technology (NTNU), Larsgårdsvegen 2, Ålesund, 6009, Norway; Health Systems Research Unit, South African Medical Research (SAMRC), Francie van Zijl Drive, Parrowvallei, Cape Town, 7503, South Africa; Health Systems Research Unit, South African Medical Research (SAMRC), Francie van Zijl Drive, Parrowvallei, Cape Town, 7503, South Africa; African Social Sciences Unit of Research and Evaluation (ASSURE), Faculty of Health Sciences, University of the Witwatersrand, 1 Jan Smuts Avenue, Braamfontein, Johannesburg, 2001, South Africa; Perinatal HIV Research Unit (PHRU), Faculty of Health Sciences, University of the Witwatersrand, 1 Jan Smuts Avenue, Braamfontein, Johannesburg, 2001, South Africa; Department of Psychology, School of Human and Community Development, University of the Witwatersrand, 1 Jan Smuts Avenue, Braamfontein, Johannesburg, 2001, South Africa; Health Systems Research Unit, South African Medical Research (SAMRC), Francie van Zijl Drive, Parrowvallei, Cape Town, 7503, South Africa; African Social Sciences Unit of Research and Evaluation (ASSURE), Faculty of Health Sciences, University of the Witwatersrand, 1 Jan Smuts Avenue, Braamfontein, Johannesburg, 2001, South Africa; Health Systems Research Unit, South African Medical Research (SAMRC), Francie van Zijl Drive, Parrowvallei, Cape Town, 7503, South Africa; Health Systems Research Unit, South African Medical Research (SAMRC), Francie van Zijl Drive, Parrowvallei, Cape Town, 7503, South Africa; Department of Paediatrics and Child Health, University of Cape Town, Lovers Walk, Rondebosch, Cape Town, 7700, South Africa; School of Public Health, University of the Western Cape, Robert Sobukwe Road, Bellville, Cape Town, 7535, South Africa

**Keywords:** mobile phone, messaging applications, healthcare workers, South Africa, informal, communication, survey

## Abstract

Mobile health (mHealth) technologies are important for supporting health service delivery and enhancing workforce performance in low- and middle-income countries. Alongside mHealth technologies endorsed by Departments of Health, healthcare workers are using messaging applications (apps), such as WhatsApp, informally on their personal mobile phones to fulfil work tasks. Our objective was to explore the use of mobile phone messaging apps for daily work among healthcare workers and managers in South Africa. We conducted a cross-sectional anonymous online survey with public and private sector healthcare workers, recruited via the Health and Welfare Sector Education and Training Authority database from February 2022 to May 2022. A total of 2174 healthcare workers participated and data were analysed using Stata SE. Most participants were female (73.8%), Caucasian (70.4%), English-speaking (53.7%), and had a postgraduate degree (58%). Over two-thirds of participants (67.5%) worked in the private sector, 81.4% were urban-based and doctors were the largest group (35%). Most participants (90%) reported using a messaging app for work-related communication, mostly WhatsApp (84%). Participants reported using messaging apps for contacting colleagues (52.1%), patient referrals (50.6%) and clinical management information (47.5%). Apps were also used for sharing appointment times and reminders (44.7%) with patients. App use across the public and private sectors and healthcare facility types was similar. Most healthcare workers (79.3%) did not receive a mobile phone data allowance. The survey shows that informal use of messaging apps by healthcare workers in South Africa is widespread and needs to be considered in digital health strategies.

## Graphical Abstract

**Graphical Abstract:**
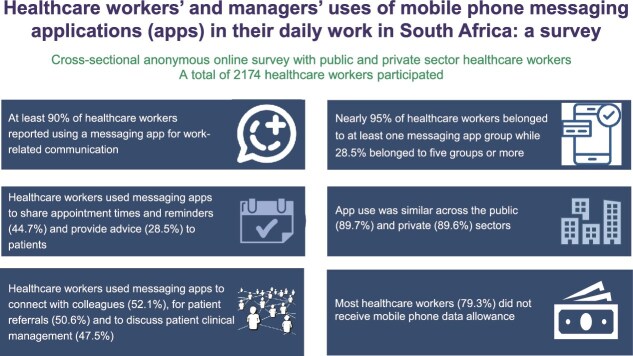

## Introduction

Digital technologies are important tools for supporting health service delivery and improving the performance of the health workforce in low- and middle-income countries (LMICs) (WHO 2017). It has been argued, however, that in many settings the potential of formal, ‘top down’ mobile health (mHealth) interventions—such as mHealth applications (apps) to manage medicines or patient referrals—has not always been realized and that there are significant scaling up challenges (Hampshire et al. 2021; WHO 2019; Anstey Watkins et al. 2018). Alongside these formal implementations of mHealth apps, healthcare workers are using their own mobile phones and free-to-download, accessible internet-based mobile phone messaging apps (referred to here as ‘mobile phone messaging apps’) *informally* to help address daily work challenges. For instance, they may use mobile phone messaging apps to contact colleagues in adjacent health facilities to obtain clinical advice or to see if a medicine is available (Alwy Al-Beity et al. 2020; Munabi-Babigumira et al. 2019). In previous work, we defined such informal mobile device use as ‘healthcare workers’ use of mobile phones and other mobile devices to support their work, using approaches that are *initiated* by the healthcare workers themselves, and that are *not initially standardized, regulated, or endorsed* by the health system or organisation to which they belong.’ ([Glenton et al. 2024] p13–14).

Recent estimates suggest that ~66% of individuals in Africa own a mobile phone and that 94% of people have at least some mobile network coverage (ITU n.d.). Mobile phone access, including smartphone access, is likely to be much higher among healthcare workers in African countries, and many have access to phone messaging apps. In many LMIC contexts, healthcare workers use their personal mobile phones and funds to purchase airtime and data for both formal and informal work uses (Glenton et al. 2023). Informal uses of mobile phone messaging apps, such as WhatsApp, grew rapidly during the COVID-19 pandemic (Jahromi and Ayatollahi 2023).

The informal digital networks facilitated by mobile messaging apps offer healthcare workers a familiar, quick and easy way to communicate and share information, for instance with colleagues in similar roles or geographic areas. Such informal networks could include all the health facility managers within a health district or all the staff working in a primary care facility. They are informal in that they are not governed or approved by the healthcare organisation that these workers are part of (Glenton et al. 2024). However, informal use raises several challenges. A recent systematic review of qualitative studies, including eighteen from LMICs, explored the views, experiences, and practices of healthcare workers, managers and other professionals regarding their informal uses of mobile devices to support their work (Glenton et al. 2024). This review found that healthcare workers are using their own mobile phones, data and airtime to bridge gaps in the resources available to support their work. Personal phone use has become routinised in some settings, with some healthcare workers feeling pressure from colleagues or managers to use their own devices at work or feeling that they should do so out of responsibility towards patients and colleagues. While informal use can improve responsiveness to patients’ needs and communication with colleagues, these approaches can also reinforce hierarchies and inequities among healthcare workers. In addition, the associated data and airtime costs for healthcare workers may be a particular burden for those on lower salaries. Informal use can, in addition, create challenges in relation to the privacy and confidentiality of patient information and may disadvantage some patient groups—for instance, those with lower digital literacy levels. Finally, these informal systems can undermine official health information systems by diverting data away from formal channels, creating gaps in record-keeping (Glenton et al. 2024).

In South Africa, the National Department of Health’s Digital Health Strategy recognizes the importance of information and communication technologies (ICT) to strengthening the health system and transforming service delivery (National Department of Health 2019). 78% of South Africans own a mobile phone (data from 2019) and, as of 2022, South Africa had 167 mobile phone subscriptions per hundred inhabitants, compared to 89 per hundred inhabitants in Sub-Saharan Africa as a whole. Additionally, 75% of South African households have internet at home (data from 2022) (ITU 2024) and smartphone penetration is over 90% (data from 2019) (ICASA 2020). In this context, the Department has initiated major mHealth interventions including MomConnect (National Department of Health 2025), NurseConnect (Nombakuse et al. 2022) and the ‘Road to Health’ app (Side-by-Side 2025), to provide information to mothers, caregivers and healthcare workers. Alongside these formal systems, healthcare workers and managers in South Africa also appear to be using mobile phone messaging apps widely as ‘workarounds’ to address day-to-day work challenges (Kauta et al. 2020; Martinez et al. 2018; Meyer et al. 2021; Kapepo et al. 2022; Morris et al. 2022). However, we lack a detailed understanding of the extent of this use nationally, variation across healthworker cadres and settings, and the range of use purposes. This study addresses this gap by surveying the use of messaging apps among healthcare workers and managers in South Africa. A better understanding of the purposes for which these cadres are using messaging apps informally may also help to identify the communication gaps that formal health management information systems (HMIS) platforms are not addressing adequately.

The objective of this study was to explore the use of mobile phone messaging apps for daily work among healthcare workers and managers in South Africa, including the range of use purposes and variation across health worker cadres and settings.

## Materials and methods

The methods are reported according to the Strengthening the reporting of observational studies in epidemiology (STROBE) guidelines for observational studies (von Elm et al. 2007).

### Research design

We conducted a prospective cross-sectional online survey with healthcare workers and managers.

### Study population

Eligible participants included those 18 years and older who were included in the database of the Health and Welfare Sector Education and Training Authority (HWSETA) as healthcare workers in the private or public sectors in South Africa. The HWSETA is the statutory body responsible for education, training and skills development for the health, social development and veterinary sectors in South Africa. Its scope of coverage includes a very wide range of healthcare workers (HWSETA 2016) and healthcare workers join the database through individual, voluntary sign-up. SETAs operate under the oversight of the South African Department of Higher Education and Training and are governed by specific legislation and regulations.

### Participant recruitment

To recruit participants, an online survey link invitation to participate in the study was shared through the HWSETA between February and May 2022. The database includes contact details for ~50 000 healthcare workers, including healthcare workers in managerial roles.

### Procedures

An anonymous online survey was designed on the RedCap survey platform. The survey was available in English. Following ethical approval, the link to the online survey was shared by a staff member of the HWSETA with healthcare workers on their database. The HWSETA shared the link with ~50 198 private and public sector healthcare workers. These healthcare workers included doctors, health facility managers, nurses, pharmacists, physiotherapists and psychologists.

### Study measures

Our study measures on mobile phone messaging app use were incorporated into a wider survey, the main purpose of which was to investigate COVID-19 vaccine hesitancy among healthcare workers. The wider survey included questions on socio-demographic characteristics. For the present study we included six questions on mobile phone messaging app use, as follows (the full questions and response options are available in Supplementary Table 1):

- Which mobile phone messaging applications do you use to communicate with other healthcare providers, with healthcare managers or with service users?
- On average, how often do you use these mobile phone messaging apps for this kind of communication?
- How many work-related messaging groups (i.e. groups in which messages are sent to multiple people) do you belong to?
- What type/s of work-related communication with healthcare providers and managers do you mainly use your mobile phone messaging app for?
- What type/s of work-related communication with patients do you mainly use your mobile phone messaging app for?
- Does your employer provide you with a cell-phone data allowance?

The survey focused on messaging app use and was not designed to explore wider issues such as the governance of the use of mobile phone apps, privacy considerations in relation to patient data or the impacts of messaging app use on patient workflows or the day-to-day work of healthcare workers. Our six key questions were also designed with a view to having a manageable total number of items within the wider COVID-19 survey.

To our knowledge, there is no validated tool for exploring messaging app use among healthcare workers. We used two main processes to develop the survey questions: firstly, we drew on unpublished findings from a rapid scoping review of qualitative studies on the use of mobile phone app messaging services by health workers and managers in LMICs (Bortoli et al. 2021). These studies highlighted a wide range of use purposes. We were able to draw on on these findings when designing response options for the items on the type/s of work-related communication - with healthcare providers and managers, and with patients - for which healthcare workers use mobile phone messaging apps. Secondly, we discussed possible questions and response options within the research team and created a first draft of the survey items. We piloted the draft questions with a small number of respondents (not included in the final dataset). In addition, the full draft survey was piloted with a small number of healthcare workers in South Africa (not included in the final dataset). Based on the pilot responses, we further revised the survey questions.

### Statistical analysis

The dataset was transferred from REDCAP to STATA SE (StataCorp 2023) for analysis. Descriptive frequencies and percentages were generated for participant demographics. The dichotomous main outcome variable ‘any app use’ was generated which included any use of a mobile phone messaging app. Descriptive analysis was undertaken comparing demographic and other explanatory variables (health professional cadre, geographic location, public or private sector) with any use of a mobile phone messaging app. We also present the purposes for which the messaging apps were used stratified by health professional category, geographic location and public or private sector. We used the chi-squared test for trend to explore associations between messaging app use and explanatory variables, with a significance threshold of *P* < 0.05.

## Results

### Description of survey participants

#### Survey response rate

A total of 48 819 healthcare workers in the HWSETA database were invited to participate in the survey. Of these, 2340 workers were enrolled and started the survey with 2167 (4.4% of those invited) completing the survey questions relating to mHealth, with some variation in the number of participants completing each question. A total of 153 participants (0.31% of those invited) were excluded from the study due to incomplete data entry.

#### Overall demographic characteristics

The majority of participants were female (73.8%; n = 1599), reported being of Caucasian origin (70.4%; n = 1526) and spoke English (53.7%; n = 1163) in their home (Table 1). Most had a postgraduate degree (58%) as their highest level of education, with medical doctors being the largest group of participants by current job title (35%). Survey participants were drawn from both the private (80.2%; n = 1738) and public sectors and from a range of healthcare facilities, with the largest group of participants working in private sector medical practice (36.6%) and based in urban areas (81.4%).

**Table 1:** Demographic characteristics of survey participants (n (%)).

**Demographic variable**	**N (%)**
Gender	
Female	1599 (73.8)
Male	565 (26.1)
Other	3 (0.1)
What is your racial or ethnic group?	
Asian	23 (1.1)
Black	275 (12.7)
Coloured	75 (3.5)
Indian	165 (7.6)
White	1526 (70.4)
Other	14 (0.6)
Prefer not to answer	89 (4.1)
Which language is mostly spoken in your home?	
Afrikaans	755 (34.8)
English	1163 (53.7)
IsiNdebele	4 (0.2)
isiXhosa	47 (2.2)
isiZulu	51 (2.4)
Sepedi	30 (1.4)
Sesotho	24 (1.1)
Setswana	36 (1.7)
Siswati	2 (0.1)
Tshivenda	14 (0.6)
Xitsonga	19 (0.9)
Prefer not to respond	22 (1.0)
What is the highest level of education you have completed?	
Matric	11 (0.5)
Post-Matric Diploma	257 (11.9)
Bachelor’s Degree	629 (29.0)
Postgraduate Degree	1261 (58.2)
Other	9 (0.4)
What is your current job title?	
Allied health professional (physiotherapist, occupational therapist, speech and hearing therapist, optometrist, dietician)	479 (22.1)
Doctor	763 (35.2)
Facility, district or institution manager	64 (3.0)
Mental health professionals (social worker, counselor, psychologist)	271 (12.5)
Nurse	342 (15.8)
Pharmacist	145 (6.7)
Other [academic at universities and Health technologists (radiographer, clinical technologist, laboratory technician)]	103 (4.8)
What sector/level of healthcare facility do you work in?	
Public sector primary healthcare clinic	49 (2.3)
Public sector community health centre	20 (0.9)
Public sector tertiary-level teaching hospital	219 (10.1)
Public sector district hospital	66 (3.0)
Private sector hospital	351 (16.2)
Private sector pharmacy	118 (5.4)
Private sector medical practice	794 (36.6)
Private sector primary care clinic	97 (4.5)
Private sector complementary and alternative medicine practice	103 (4.8)
Non-Governmental Organization (NGO)	43 (2.0)
Other	307 (14.2)
Please select the area where your health facility is based	
Rural	214 (9.9)
Urban	1764 (81.4)
Peri-urban	189 (8.7)

### Demographic status and app use

Overall, 90% of participants reported using a mobile phone messaging app for work-related communication. The most common app used was WhatsApp (84%) with the remaining participants using Telegram (8%), Facebook Messenger (5%), or Signal (3%).

Participants from all educational backgrounds reported high mobile phone messaging app use, with a trend towards more use with higher levels of education although these differences were not large (*P* = 0.08; Table 2). These data may in part reflect the way in which the survey was distributed, which was largely electronic, as well as the private sector employment of most participants.

**Table 2:** Messaging app use and educational background (n (%)).

	**Matric (secondary school leaving examination)**	**Post-matric diploma**	**Bachelor’s degree**	**Postgraduate degree**	**Other**	**chi-square p-value**
Do not use messaging apps	2 (18.2)	36 (14.0)	74 (11.8)	113 (9.0)	1 (11.1)	0.08
Any messaging app use (combined data across app categories)	9 (81.8)	221 (86.0)	555 (88.2)	1148 (91.0)	8 (88.9)	

Most survey participants were nurses or doctors (51%) while the remaining participants were allied health professionals, mental health professionals or indicated another role. Messaging app use by participants’ current job title or cadre shows very high use across all cadres, including nurses and doctors (Table 3).

**Table 3:** Messaging app use and cadre (based on current job title) (n (%)).

	**Doctor**	**Nurse**	**Allied health professional** ^*^	**Mental healthcare worker** **^†^**	**Facility/district/institution manager**	**Pharmacist**	**University academic**	**Health technologist** **^‡^**	**Other healthcare worker**	**chi-square p-value**
Do not use messaging apps	63 (8.3)	35 (10.2)	45 (9.4)	31 (11.4)	9 (14.1)	27 (18.6)	4 (16.7)	12 (16.2)	0 (0.0)	<0.001
Any messaging app use (combined data across app categories)	700 (91.7)	307 (89.8)	434 (90.6)	240 (88.6)	55 (85.9)	118 (81.4)	20 (83.3)	62 (83.8)	5 (100.0)	

^*^Physiotherapists, occupational therapists, dieticians, speech and hearing therapists, optometrists; ^†^Social workers, counselors, psychologists; ^‡^Radiographers, clinical technologists, laboratory technicians

App use was similar across participants based in the public and private sectors (89.7% and 89.6% respectively, *P* = 0.95; Table 4) and we also saw similar levels of app use across all types of health care facilities (community health centres, district hospitals, private hospitals, etc. (data not shown)). App use was also similar across different geographic places of work (rural, peri-urban or urban) (*P* = 0.40; Table 5). However, relatively few survey participants worked in rural and peri-urban settings (n = 403 combined).

**Table 4:** Messaging app use across public and private sector employment (n (%))^*^

	**Private sector employment**	**Public sector employment**	**chi-square p-value**
Do not use messaging apps	181 (10.4)	44 (10.3)	0.95
Any messaging app use (combined data across app categories)	1557 (89.6)	383 (89.7)	

^*^The total number of participants who answered this question was 2165

**Table 5:** Messaging app use and geographic place of work (n (%)).

	**Rural**	**Urban**	**Peri-urban**	**chi-square p-value**
Do not use messaging apps	23 (10.8)	178 (10.1)	25 (13.2)	0.4
Any messaging app use (combined data across app categories)	191 (89.3)	1586 (89.9)	164 (86.8)	

### Frequency and type of app use

We asked participants to indicate how often, on average, they use mobile phone messaging apps for work-related communication. Close to half of participants (48.0%) used these apps more than once a day while two thirds of participants (67.3%) used apps at least once a day (Table 6). There were no important differences in level of use across health cadres (*P* = 0.07).

**Table 6:** Frequency of messaging app use and current job title (cadre) (n (%)).

**Messaging app use**	**Doctor**	**Nurse**	**Allied health professional** ^*^	**Mental healthcare worker** **^†^**	**Facility/district/institution manager**	**Pharmacist**	**University academic**	**Health technologists** **^‡^**	**Other healthcare worker**	**chi-square p-value**
Less than once a week	72 (12.0)	37 (14.4)	65 (16.7)	44 (21.5)	12 (22.6)	12 (12.0)	2 (11.1)	6 (11.5)	1 (50.0)	0.07
Once a week	96 (16.1)	45 (17.5)	81 (20.8)	34 (16.6)	10 (18.9)	18 (18.0)	3 (16.7)	9 (17.3)	1 (50.0)	
Once a day	109 (18.2)	52 (20.2)	75 (19.3)	36 (17.6)	9 (17.0)	21 (21.0)	4 (22.2)	17 (31.7)	0 (0.0)	
More than once a day	321 (53.7)	123 (47.9)	168 (43.2)	91 (44.4)	22 (41.5)	49 (49.0)	9 (50.0)	20 (38.5)	0 (0.0)	

^*^Physiotherapists, occupational therapists, dieticians, speech and hearing therapists, optometrists; ^†^Social workers, counselors, psychologists; ^‡^Radiographers, clinical technologists, laboratory technicians

When we look at the relationship between app use and whether participants worked in the public sector or other sectors, we see that participants in the public sector were more likely to use these apps more than once a day in their work (63.3%), compared to participants from outside the public sector (44.3%) (*P* < 0.001; Table 7). There were no differences in frequency of app use across participants with different geographic places of work (rural, peri-urban or urban, *P* = 0.12; Supplementary Table 2).

**Table 7:** Frequency of messaging app use and employment in the public sector or other sectors (n (%)).

**Messaging app use**	**Employment in other sectors** ^*^	**Public sector employment~**	**chi-square p-value**
Less than once a week	220 (16.3)	31 (9.5)	<0.001
Once a week	257 (19.1)	40 (12.2)	
Once a day	274 (20.3)	49 (15.0)	
More than once a day	596 (44.3)	207 (63.3)	

^*^Includes all private health facilities: primary care clinics, medical practices, pharmacies, hospitals, alternative providers, NGOs, others; ~ Includes all public facilities: primary healthcare clinics, community health centres, district hospitals, tertiary hospitals

### Participation in work-related messaging groups

We asked participants to indicate how many work-related messaging groups (i.e. groups in which messages are sent to multiple people) they belong to. Close to 95% of participants were members of at least one messaging group while over a quarter of participants (28.5%) belonged to five groups or more. There were differences across cadres in the number of app messaging groups that participants belonged to, with doctors, nurses, pharmacists and managers belonging to more groups on average than other cadres (*P* < 0.001; Table 8).

**Table 8:** Current job title (cadre) and participation in work-related app messaging groups (n (%)).

**Number of messaging groups**	**Doctor**	**Nurse**	**Allied health professional** ^*^	**Mental health worker** **^†^**	**Facility/district/institution manager**	**Pharmacist**	**University academic**	**Health technologist** **^‡^**	**Other healthcare worker**	**chi-square p-value**
None	28 (4.7)	14 (5.5)	27 (7.0)	20 (9.8)	2 (3.8)	1 (1.0)	1 (5.6)	2 (3.9)	0 (0.0)	<0.001
One	57 (9.5)	25 (9.8)	55 (14.2)	36 (17.6)	7 (13.2)	20 (20.0)	2 (11.1)	7 (13.5)	0 (0.0)	
Less than 5	326 (54.5)	130 (50.8)	214 (55.2)	108 (52.7)	25 (47.2)	48 (48.0)	6 (33.3)	33 (63.5)	2 (100.0)	
5–10	143 (23.97)	64 (25.0)	78 (20.1)	33 (16.1)	14 (26.4)	26 (26.0)	8 (44.4)	10 (19.2)	0 (0.0)	
More than 10	44 (7.4)	23 (9.0)	14 (3.6)	8 (3.9)	5 (9.4)	5 (5.0)	1 (5.6)	0 (0.0)	0 (0.0)	

^*^Physiotherapists, occupational therapists, dieticians, speech and hearing therapists, optometrists; ^†^Social workers, counselors, psychologists; ^‡^Radiographers, clinical technologists, laboratory technicians

We also found important differences between participants working in the public sector and those in other sectors regarding the number of work-related messaging groups they belonged to, with public sector workers more likely to participate in five or more messaging groups (*P* < 0.001; Table 9). There were no differences in level of participation in messaging groups across participants with different geographic places of work (rural, peri-urban or urban, *P* = 0.42; Supplementary Table 3).

**Table 9:** Public or other sector employment and participation in work-related app messaging groups (n (%)).

**Number of messaging groups**	**Private health facilities** ^*^	**Public health facilities**^†^****	**chi-square p-value**
None	90 (6.7)	5 (1.5)	<0.001
One	183 (13.6)	26 (8.0)	
Less than 5	728 (54.1)	164 (50.2)	
5–10	227 (20.6)	99 (30.3)	
More than 10	67 (5.0)	33 (10.1)	

^*^Includes all private health facilities: primary care clinics, medical practices, pharmacies, hospitals, alternative providers, NGOs, others; ^†^Includes all public facilities: primary healthcare clinics, community health centres, district hospitals, tertiary hospitals

### Purposes for which messaging apps are being used to communicate with other healthcare workers

The most common purposes for using messaging apps that participants identified were: connecting to health worker colleagues in the same facility, district or province (52.1%, n = 871); discussing referral of a patient with another healthcare provider (50.6%, n = 846); asking another healthcare provider for advice regarding the clinical management of a patient (47.5%, n = 794); and sending or receiving new clinical management guidelines (43.3%, n = 724). For some messaging app use purposes, we found variation across cadres in the proportion of participants who used these (Supplementary Table 4). For instance, a smaller proportion of nurses used apps to discuss referral of a patient with another provider (30.0%, n = 92), compared to doctors (46.1%, n = 323), allied health (53.7%, n = 233) or mental health professionals (56.3%, n = 135) (*P* < 0.001). The survey did not explore reasons for this variation, but this may be because nurses have face-to-face access to doctors in their facility or because referral tasks are seen as the responsibility of doctors. Further, the majority of nurses in the survey worked in the private sector and may have had other mechanisms for discussing possible referrals.

We also found that the proportion of participants who used app groups to ask another healthcare provider for advice regarding the clinical management of a patient was similar among doctors, nurses and allied health professionals (46,7% (n = 327), 40.7% (n = 125) and 41.9% (n = 182) respectively) but lower among other cadres such as health technologists and facility managers (24.2% (n = 15) and 29.1% (n = 16) respectively, *P* < 0.001). All healthcare provider groups used messaging apps to some extent to send or receive new clinical management guidelines. However, there was important variation across cadres, with larger proportions of doctors, nurses and managers noting this use purpose, perhaps reflecting their roles in their settings (*P* < 0.001; Supplementary Table 4).

For some messaging app use purposes, we also found variation across providers employed in the public and private sectors. A higher proportion of participants in public sector facilities used apps to connect to health worker colleagues in the same facility, district or province and to send or receive new clinical guidelines, compared to in private sector facilities (*P* < 0.001; Table 10). This is perhaps because public sector facilities are part of a district infrastructure or because these providers lack other reliable digital mechanisms for these purposes, such as email. In contrast, the proportions were similar for referring patients to another healthcare provider and asking another provider for advice regarding the clinical management of a patient.

**Table 10:** Purposes for which messaging apps are used in the workplace to communicate with other healthcare providers and managers, by public or other sector employment (n (%)).

**Purpose** **^#^**	**Private health facilities** ^*^	**Public health facilities** **^†^**	**chi-square p-value**
Connect to health worker colleagues in the same facility/district/province	666 (42.8) ~	201 (52.5)	<0.001
Discuss referral of a patient with another healthcare provider	684 (43.9)	158 (41.3)	0.34
Ask another healthcare provider for advice regarding the clinical management of a patient	636 (40.9)	154 (40.2)	0.82
Send or receive new clinical management guidelines	541 (34.8)	178 (46.5)	<0.001

^#^Participants could select more than one option. ‘Other purposes’ constituted 2.6% (n = 43) of responses—these are not shown in the table; ^*^Includes all private health facilities: primary care clinics, medical practices, pharmacies, hospitals, alternative providers, NGOs, others; ^†^Includes all public facilities: primary healthcare clinics, community health centres, district hospitals, tertiary hospitals; ~ For type of sector, the remaining participants indicated that they did not use their phone for this type of communication

Variation in the use of messaging apps was also found across the workplace settings of participants. There were indications of a trend towards more use of apps to connect with health worker colleagues from rural to urban and peri-urban (38.2% (n = 73), 45.0% (n = 713) and 49.4% (n = 81) respectively; *P* = 0.08), but little or no difference across these settings in relation to the proportion of participants using apps to discuss the referral of patients, to ask for advice regarding clinical management or to send or receive new guidelines (Supplementary Table 5).

### Purposes for which messaging apps are being used to communicate with patients

In relation to communicating with patients, the most common purposes that participants identified included: sharing appointment times and reminders with patients (44.7%, n = 867); providing advice to patients on their health issue (28.5%, n = 553); and sharing information with patients (20.7%, n = 402). For all patient communication purposes explored in the survey, we found important variation across cadres in the proportion of participants using messaging apps for these. For example, much higher proportions of physiotherapists and psychologists used apps to share appointment times and reminders with patients, compared to the other cadres (*P* < 0.001). This may be because more of these healthcare workers are in private practice and see each patient for a larger number of sessions over time. Overall, messaging apps were not used extensively to contact patients who had defaulted from treatment (16.4%, n = 318) or to send test results to patients (14.7%, n = 286 providers), with 72.3% (n = 207) of the latter being done by doctors (Table 11).

**Table 11:** Purposes for which messaging apps are used to communicate with patients, by current job title (n (%)).

**Purpose** **^#^**	**Doctor**	**Nurse**	**Allied health professional** ^*^	**Mental health worker** **^†^**	**Facility/district/institution manager**	**Pharmacist**	**University academic**	**Health technologist** **^‡^**	**Other**	**chi-square p-value**
Share appointment times and reminders with patients	208 (29.7) ~	114 (37.1)	296 (68.2)	180 (75.0)	19 (34.6)	22 (18.6)	5 (25.0)	22 (35.5)	1 (20.0)	<0.001
Contact patients who have defaulted on treatment	85 (12.1)	56 (18.2)	102 (23.5)	37 (15.4)	10 (18.2)	19 (16.1)	1 (5.0)	7 (11.3)	1 (20.0)	<0.001
Send test results to patients	207 (29.6)	42 (13.7)	20 (4.6)	2 (0.8)	3 (3.4)	4 (3.4)	1 (5.0)	7 (11.3)	0 (0.0)	<0.001
Provide advice to patients on their health issue	211 (30.1)	108 (35.2)	132 (30.4)	32 (13.3)	16 (29.1)	42 (35.6)	4 (20.0)	8 (12.9)	0 (0.0)	<0.001
Share information with patients	112 (16.0)	79 (25.7)	115 (26.5)	40 (16.7)	11 (20.0)	29 (24.6)	7 (35.0)	9 (14.5)	0 (0.0)	<0.001
Other	162 (23.1)	62 (20.2)	46 (10.6)	14 (5.8)	16 (29.1)	26 (22.0)	9 (45.0)	17 (27.4)	1 (20.0)	<0.001

^#^Participants could select more than one option. ‘Other purposes’ constituted 2.6% (n = 43) of responses—these are not shown in the table; ^*^Physiotherapists, occupational therapists, dieticians, speech and hearing therapists, optometrists; ^†^Social workers, counselors, psychologists; ^‡^Radiographers, clinical technologists, laboratory technicians; ~ For each cadre, the remaining participants indicated that they did not use their phone for this type of communication

For all purposes except contacting patients who had defaulted from treatment, the use of messaging apps to communicate with patients was much more common among healthcare workers based in private health facilities of various kinds, compared to publicly funded facilities (Supplementary Table 6). For example, 49.2% (n = 766) of workers in private facilities used messaging apps to share appointment times and reminders with patients, compared to 26.4% (n = 101) in public facilities (*P* < 0.001). This may reflect higher access to smartphones and data among healthcare workers in private facilities or differing expectations around patient communication.

For some communication purposes, the use of apps varied depending on whether the healthcare provider was based in a rural, urban or peri-urban facility (Supplementary Table 7). For example, healthcare workers in urban and peri-urban facilities more frequently used messaging apps to share appointment times and reminders with patients (46.8% [n = 742] and 37.8% [n = 62] respectively), compared to those in rural facilities (33.0% [n = 63]; *P* < 0.001). There was little geographic variation, however, in relation to contacting patients who have defaulted from treatment or sharing information with patients. Reasons for these patterns were not explored in the survey.

### Allowances to support mobile phone use by healthcare workers

Most healthcare workers reported that they did not receive a mobile phone data allowance from their employer to support use of their personal mobile phone for work purposes (79.3%, n = 1497). However, there was important variation across workers, with facility managers most frequently reporting receiving an allowance (37.3%, n = 22) and pharmacists (11.3%, n = 14) least frequently doing so (*P* < 0.001; Table 12). Further, healthcare workers in private facilities were almost twice as likely to receive an allowance compared to those working in publicly funded facilities (22.7% (n = 344) compared to 12.7% (n = 47); *P* < 0.001, Supplementary Table 8). While healthcare workers in rural (26.2%, n = 47) and peri-urban (25%, n = 42) areas more frequently reported receiving an allowance compared to those working in urban facilities (19.6%, n = 302), these differences were smaller than between public and private facilities\(*P* = 0.04; Supplementary Table 8).

**Table 12:** Healthcare workers’ receipt of a mobile phone data allowance from their employer, by current job title (n (%)).

	**Doctor**	**Nurse**	**Allied health professional** ^*^	**Mental health worker** **^†^**	**Facility or district or institution manager**	**Pharmacist**	**University academic**	**Health technologist** **^‡^**	**Other**	**chi-square p-value**
Receive an allowance	122 (18.5)	60 (20.8)	96 (22.2)	51 (21.6)	22 (37.3)	14 (11.3)	3 (13.6)	21 (32.8)	2 (100.0)	<0.001
Do not receive an allowance	536 (81.5)	229 (79.2)	336 (77.8)	185 (78.4)	37 (62.7)	110 (88.7)	19 (86.4)	43 (67.2)	0 (0.0)	

^*^Physiotherapists, occupational therapists, dieticians, speech and hearing therapists, optometrists; ^†^Social workers, counselors, psychologists; ^‡^Radiographers, clinical technologists, laboratory technicians

## Discussion

This was a large survey of healthcare workers in South Africa to explore their informal use of messaging apps to support their daily work. We found high use of mobile phone messaging apps, almost exclusively WhatsApp, amongst healthcare workers. This usage was found to be equally high amongst public and private sector participants with two thirds of participants using this app at least once a day. Almost all participants belonged to at least one messaging group with the main purposes of app use being to connect with other colleagues, to discuss referrals, to seek clinical advice or to share clinical guidelines. Healthcare workers also reported using messaging apps to communicate with patients for the purposes of sharing appointments and reminders, especially amongst allied health and mental health professionals. A striking finding was that despite the high use of messaging apps, most healthcare workers did not receive a data allowance from their employer, particularly those working in the public sector. While the survey focused specifically on messaging app use and did not explore broader issues such as the impacts of this use on patient data privacy or the organization of day-to-day work, it provides useful insights regarding the scope of messaging app use by South African healthcare workers.

The growth of mobile digital technology globally has enabled increased access to apps for individual and group communication. Global health implementation partners and research funding have focused largely on ‘formalized’ and employer-approved mHealth technologies for healthcare workers (Hampshire et al. 2021). However, these formal approaches do not necessarily consider the ways in which healthcare workers and service users want or need to communicate with one another, and ways of making that communication ‘easy.’ The widespread informal use of mobile phone messaging apps that this study highlights suggests that healthcare workers find these easy to use but also that they plug important gaps in the formal communication systems available to healthcare workers, creating informal digital networks within health districts and more widely. A recent South African study of how healthcare workers are using formal electronic referral systems in public hospitals also showed the wide use of messaging apps as workarounds for these systems. The study suggests that this use relates to gaps between how formal digital health tools are designed and the working realities of healthcare workers. For example, healthcare workers were not involved adequately in the planning and selection of tools and so their needs were not well understood; plans for implementing the tools were not well designed; and formal IT support and internet access were poor (Kapepo 2025). A systematic review of digital technologies used during the COVID-19 pandemic in South Africa also identified significant challenges and limitations of the formal digital tools used, including lack of equipment and connectivity and poor interoperability across systems (Mbunge et al. 2022). Studies from other African countries similarly show the wide use of personal mobile phones for work-related purposes, including communication through WhatsApp, and relatively low use of formal mHealth applications (Hampshire et al. 2017), pointing to challenges with the formal digital health infrastructure.

The informal networks facilitated by messaging apps allow healthcare workers to contact colleagues about referrals or for clinical advice, to share reminders and advice with patients and to undertake a range of other tasks. This wide range of informal uses may have both positive and negative impacts on workflows within and across health facilities as well as on the timeliness and quality of patient care in LMIC settings. For instance, being able to discuss patient referrals with another healthcare provider may increase the clinical appropriateness of referrals and help ensure that patients are directed to a suitable referral facility (Kapepo 2025). Informal use may also make health services more responsive to people’s needs and more efficient. Table 13 outlines key future research questions to explore these and other potential impacts.

**Table 13:** Future research questions on informal uses of mobile messaging apps by healthcare workers.

What are the impacts of the informal use by healthcare workers of messaging apps, and of personal mobile phones more broadly, on equity of information access, workflows within and across health facilities and on the timeliness and quality of patient care? To what extent do healthcare workers’ informal uses of mobile phone messaging apps lead to health services that are more responsive to the needs of patients and their caregivers? In what ways do healthcare workers’ informal uses of mobile phone messaging apps, and personal mobile phone use more broadly, blur work/home boundaries? What governance strategies can be put in place by service delivery organisations to manage the potential downsides of informal messaging app use, such as breaches of patient confidentiality and loss of key management information from the health system? What strategies can be used to address equitably the financial burden on healthcare workers of personal mobile phone use? In what ways can national digital health strategies support the integration of messaging app use into formal digital health systems? Which survey items demonstrate validity and reliability in assessing the frequency and functional purposes of healthcare workers’ use of messaging apps and of informal mobile phone use more broadly?

Informal mobile phone use may have important downsides for healthcare workers. Our survey indicates that most healthcare workers did not receive a mobile phone data allowance from their employer to support use of their personal mobile phone for work purposes and covered these costs themselves. A qualitative evidence synthesis of healthcare worker’s informal uses of mobile phones also found that this use had cost implications for the workers in terms of use of their own personal device and data and that these costs can be a significant financial burden for low salaried staff and community workers (Glenton et al. 2024). Similarly, a qualitative study in rural Mpumalanga, South Africa exploring mobile phone use amongst patients and healthcare workers in primary care settings found both that this bottom-up use was widespread and that the cost of personal phone use was carried by the healthcare workers themselves (Anstey Watkins et al. 2018). Our survey did not explore the reasons why most participating healthcare workers did not receive reimbursement for costs related to their personal mobile phone use, but this may relate to difficulties in reimbursing costs for uses that are not formally approved or regulated and administrative challenges in applying for or distributing data allowances, particularly in the public sector. This hypothesis is supported by our data showing that healthcare workers in private facilities were almost twice as likely to receive an allowance, compared to those working in publicly funded facilities. In addition to the financial burden of informal use, studies have also noted threats to the personal wellbeing of healthcare workers, linked to disturbance during and after work and the blurring of work/home boundaries (Glenton et al. 2024; Hampshire et al. 2017).

In our survey, healthcare workers reported using WhatsApp and other messaging apps for multiple purposes including to distribute guidelines and get advice from colleagues. Keeping health professionals up to date as clinical protocols and guidelines change is a vital health system responsibility. However, reaching community and primary care staff and those in rural areas can be particularly challenging as these staff cannot easily attend ongoing professional education opportunities at academic hospitals and may not have regular contact with specialists (Ramsden et al. 2022; Ramsden and Lincoln 2022). Informal use of messaging apps appears to be filling a gap not fully addressed by formal health sector communication channels and has the potential to improve the equity of information access. Other studies have shown similar findings: an evaluation of a WhatsApp group connecting primary care doctors with orthopaedic surgeons in the Western Cape Province of South Africa found that this information communication method was effective in enabling support to non-specialists for fracture management (Kauta et al. 2020). Also, a descriptive study in the Eastern Cape Province of the roles of a WhatsApp group as a learning tool to improve clinicians’ access to specialized management of complicated HIV/TB cases found that group participants gained new clinical confidence. This was associated with posting of questions, new clinical insights and the use of peer guidance to manage cases (Woods et al. 2019).

Our research also highlights important support needs for front line healthcare providers. Currently the informal use of mobile messaging apps, and the time and costs involved, is largely ‘invisible’ in national and provincial policy documents on the roles of digital or mHealth interventions in the South Africa health care system—e.g. the National Digital Health Strategy does not explicitly consider these informal uses (National Department of Health 2019; Verdezoto et al. 2021; Bergey et al. 2019). Our study suggests that national digital health strategies need to consider the informal use of mobile messaging apps, including how best to provide infrastructure and resources, such as Wi-Fi access, to support this use and guidance on the appropriate use of messaging groups as clinical support tools. Health systems also need to consider how to address the challenge of different levels of digital literacy and smartphone access among both healthcare workers and patients as well as how messaging app use should be governed, to ensure the confidentiality of patient information and reduce the loss of key management information from the health system.

This study had several strengths and limitations. Strengths include the large sample size that covered a wide diversity of health worker cadres across both private and public sectors and both rural and urban settings in South Africa. A limitation of the survey process is that respondents were mostly English speaking, Caucasian, female healthcare workers with postgraduate qualifications. Further, a large proportion of survey participants were working in urban settings and in private sector health care. Public sector healthcare workers, managers and rural healthcare workers, as well as healthcare workers with other language and ethnic group backgrounds, are therefore relatively under-represented in the data. The reasons for this are unclear but may relate to our choice of an online survey approach, including for recruitment of participants, which may have decreased access for public sector and rural healthcare workers and managers. It is also possible that the online recruitment method may have led to the survey overestimating messaging app use. However, ownership of mobile phones is very high in the South African population (ITU 2024), and likely to be even higher among South African healthcare workers, so this bias may not be large.

The database of the HWSETA, through which survey participants were recruited, is populated through individual sign-up by healthcare workers. It is unclear to what extent this database is representative of all healthcare workers in South Africa. Further, the relatively low survey response rate means that non-response bias cannot be excluded. These aspects may limit the generalizability of the findings to all healthcare workers in South Africa although we found no important differences in frequency of app use or membership of messaging app groups across participants with different geographic places of work.

Finally, the survey tool had some limitations. As we were not aware of any validated tools in this area, we developed and piloted the survey questions ourselves and it is possible that a more extensive validation process would have improved the reliability of the questions—this is a potential area for future research (Table 13). Also, the survey did not explore wider concerns such as how mobile phone app use is governed, privacy considerations in relation to patient data or healthcare workers’ experiences of informal mobile messaging app use. While we recognize the importance of these questions, our aim here was to gather a broad picture of the extent and pattern of app use. Healthcare workers’ experiences of app use was the focus of a separate qualitative study that will be reported elsewhere.

## Conclusion

A large proportion of healthcare workers in South Africa use messaging apps daily across both public and private sectors, healthcare worker types and settings. These apps support core functions such as sharing information, discussing patient referrals, seeking clinical advice, sharing appointment times with and advising patients. The widespread informal use of messaging apps reflects both their convenience and wide penetration but also gaps within formal communication systems. Most healthcare workers, especially in the public sector, bear the financial cost without support. We are exploring these issues further through qualitative research in India, South Africa and Uganda (Glenton et al. 2023).

Other studies suggest that healthcare workers often perform invisible ‘maintenance’ work to keep health systems running, creating digital workarounds when formal digital systems fall short (Verdezoto et al. 2021; Bergey et al. 2019). Our findings support this perspective indicating that informal uses of mobile messaging apps are filling important gaps. Health systems should invest in infrastructure that supports these ‘bottom-up’ communication methods and further research is needed on ways to integrate informal messaging app use into formal digital health systems (Glenton et al. 2024) (Table 13).

## Supplementary Material

oqag005_mHEALTH_INNOVATE_survey_paper_SUPPLEMENT_TABLES_2026_02_04
