# Supplementary material for: Healthcare workers’ and managers’ uses of mobile phone messaging applications (apps) in their daily work in South Africa: a survey
Source: Oxf Open Digit Health. 2026 Feb 17;4:oqag005. doi: 10.1093/oodh/oqag005 (PMC12962228; doi:10.1093/oodh/oqag005)
Supplement: oqag005_mHEALTH_INNOVATE_survey_paper_SUPPLEMENT_TABLES_2026_02_04 [file oqag005_mhealth_innovate_survey_paper_supplement_tables_2026_02_04.pdf]

Healthcare workers' and managers' uses of mobile phone messaging applications (apps) in their daily work in South Africa: a survey

Supplementary tables

Supplementary table 1: Online Survey for Healthcare Workers – demographic questions and questions on use of mobile phone messaging apps

	Domain	Question
1	**Demographics**	What is your age?
2	**Demographics**	What is your gender?<br>☐1 Female<br>☐2 Male<br>☐3 Other, specify: ______________________
3	**Demographics**	What is your racial/ethnic group?<br>☐1 Black<br>☐2 Asian<br>☐3 Coloured<br>☐4 White<br>☐5 Other, specify: ______________________<br>☐6 Prefer not to respond
4	**Demographics**	Which language is mostly spoken in your home?<br>☐1 isiZulu<br>☐2 isiXhosa<br>☐3 Afrikaans<br>☐4 SeSotho<br>☐5 Tshivenda<br>☐6 Xitsonga<br>☐7 Sepedi<br>☐8 English<br>☐9 Setswana<br>☐10. siNdebele<br>☐11 siSwati<br>☐12 Prefer not to respond
5	**Demographics**	What is the highest level of education you have completed?<br>☐1 Matric

		☐2 Post-matric diploma<br>☐3 Bachelor's degree<br>☐4 Post-graduate degree<br>☐5 Other, specify: ___________________________________
6	**Demographics**	What is your current job title?<br><br>☐1 Doctor<br>☐2 Nurse<br>☐3 Physiotherapist<br>☐4 Psychologist<br>☐5 Facility manager<br>☐6 Pharmacist<br>☐7 Other, specify:<br>____
7	**Demographics**	What sector/level of healthcare facility do you work in?<br><br>☐1 Public sector primary healthcare clinic<br>☐2 Public sector community health center<br>☐3 Public sector tertiary-level teaching hospital<br>☐4 Public sector district hospital<br>☐5 Private sector hospital<br>☐6 Private sector pharmacy<br>☐7 Private sector medical practice<br>☐8 Private sector primary care clinic<br>☐9. Private sector complementary and alternative medicine practice<br>☐10 Non-Governmental Organization (NGO)<br>☐11 Other, specify: ____
8		Please select the area where your health facility is based.<br>☐1 Rural<br>☐2 Urban<br>☐3 Peri-urban
25	**Mobile application use**	Which mobile phone messaging applications do you use to communicate with other healthcare providers, with healthcare managers or with service users? [Select all that apply]<br>1. WhatsApp<br>2. Signal<br>3. Telegram<br>4. Facebook messaging<br>5. Other (please specify)<br>**6.** I do not use mobile phone messaging **apps as part of my work**
26	**Mobile application use**	On average, how often do you use these mobile phone messaging apps for this kind of communication? [Select one]<br><br>☐1 Less than once a week<br>☐2 Once a week<br>☐3 Once a day

		☐4 More than once a day
27	**Mobile application use**	How many work-related messaging groups (i.e., groups in which messages are sent to multiple people) do you belong to? [Select one]<br><br>☐1 None<br>☐2 One<br>☐3 Less than 5<br>☐4 5-10<br>☐4 More than 10
28	**Mobile application use**	What type/s of work-related communication <u>with healthcare providers and managers</u> do you mainly use your mobile phone messaging app for? [Select all apply]<br><br>1. Ask another healthcare provider for advice regarding the clinical management of a patient<br>2. Discuss referral of a patient with another health care provider<br>3. Refer a patient to community-based services (e.g., NGOs, faith-based organisations, community health workers)<br>4. Ask another healthcare provider for advice regarding a departmental procedure (e.g., ordering new equipment or organizing repair of equipment)<br>5. Ask a healthcare manager for advice on a work-related issue<br>6. Report medicines or other commodity stockouts<br>7. Check with adjacent health facilities regarding the availability of medicines or commodities<br>8. Provide clinical advice to other health care providers<br>9. Provide emotional support to other health care providers<br>10. Send facility information to healthcare managers (for example, statistics on number of people attending for family planning, child health etc.)<br>11. Send or receive new clinical management guidelines<br>12. Connect to health worker colleagues in the same facility / district / province<br>13. Connect to health worker colleagues who work with the same health issues (e.g., HIV and AIDS, COVID-19, vaccines, TB etc.)<br>14. Connect to service providers in other sectors (e.g., SASSA, police, social workers, teachers)<br>15. Other (specify)
29	**Mobile application use**	What type/s of work-related communication with <u>patients</u> do you mainly use your mobile phone messaging app for? [Select all that apply]<br><br>1. Share appointment times and reminders with patients<br>2. Contact patients who have defaulted on treatment<br>3. Send test results to patients<br>4. Provide advice to patients on their health issue<br>5. Share information with patients (for example, on health promotion activities)<br>6. Other (specify)
30	**Mobile application use**	Does your employer provide you with cell-phone data allowance?<br><br>1. Yes

		2. No

Supplementary table 2: Frequency of messaging app use by geographic place of work (n (%))

Messaging app use	Rural	Urban	Peri-urban	chi-square p-value
Less than once a week	20 (12.99)	208 (15.13)	23 (15.86)	0.12
Once a week	22 (14.29)	255 (18.55)	20 (13.79)	
Once a day	26 (16.88)	259 (18.84)	38 (26.21)	
More than once a day	86 (55.84)	653 (47.49)	64 (44.14)	

Supplementary table 3: Geographic place of work and participation in work-related app messaging groups (n (%))

Number of messaging groups	Rural	Urban	Peri-urban	chi-square p-value
None	11 (7.14)	79 (5.75)	5 (3.45)	0.42
One	20 (12.99)	166 (12.09)	23 (15.86)	
Less than 5	71 (46.10)	738 (53.75)	83 (57.24)	
5-10	41 (26.62)	308 (22.43)	27 (18.62)	
More than 10	11)7.14)	82 (5.97)	7 (4.83)	

Supplementary table 4: Purposes for which messaging apps are used in the workplace to communicate with other healthcare providers and managers, by current job title (n (%))

**Purpose**	**Doctor**	**Nurse**	**Allied health professional***	**Mental health worker^**	**Facility / district / institution manager**	**Pharmacist**	**University academic**	**Health technologist ¤**	**Other healthcare worker**	**chi-square p-value**
Connect to health worker colleagues in the same facility / district /province	322 (46.00)~	140 (45.60)	200(46.08)	87 (36.25)	25 (45.45)	57 (48.31)	9 (45.00)	27 (43.55)	0 (0.00)	0.12
Discuss referral of a patient with another healthcare provider	323 (46.14)	92 (29.97)	233 (53.69)	135(56.25)	16 (29.09)	18 (15.25)	7 (35.00)	18 (29.03)	5 (100)	0.00
Ask another healthcare provider for advice regarding the clinical management of a patient	327 (46.71)	125 (40.72)	182 (41.94)	74 (30.83)	16 (29.09)	45 (38.14)	6 (30.00)	15 (24.19)	0 (0.00)	0.00
Send or receive new clinical management guidelines	367 (52.43)	120 (39.09)	112 (25.81)	32 (13.33)	30 (54.55)	36 (30.51)	7 (35.00)	14 (22.58)	1 (20.00)	0.00

[#] Participants could select more than one option. 'Other purposes' constituted 2.6% (n=43) of responses – these are not shown in the table; *Physiotherapists, occupational therapists, dieticians, speech and hearing therapists, optometrists; ^Social workers, counselors, psychologists; ¤Radiographers, clinical technologists, laboratory technicians; ~ For each cadre, the remaining participants indicated that they did not use their phone for this type of communication

Supplementary table 5: Purposes for which messaging apps are used in the workplace, by geographic place of work (n (%))

Purpose	Rural	Urban	Peri-urban	chi-square p-value
Connect to health worker colleagues in the same facility / district /province	73 (38.22) ~	713 (44.96)	81 (49.39)	0.09
Discuss referral of a patient with another healthcare provider	71 (37.17)	703 (44.33)	68 (41.46)	0.15
Ask another healthcare provider for advice regarding the clinical management of a patient	65 (34.03)	658 (41.49)	67 (40.85)	0.14
Send or receive new clinical management guidelines	74 (38.74)	574 (36.19)	71 (43.29)	0.17
Other	5 (2.34)	36 (2.04)	2 (1.06)	Not calculated

[#] Participants could select more than one option; ~ For each place of work, the remaining participants indicated that they did not use their phone for this type of communication

Supplementary table 6: Purposes for which messaging apps are used to communicate with patients, across public and private health care facilities (n (%))

Purpose	Private health facilities~	Public health facilities*	chi-square p-value
Share appointment times and reminders with patients	766 (49.20)^	101 (26.37)	0.00
Contact patients who have defaulted on treatment	254 (16.31)	64 (16.71)	0.85
Send test results to patients	246 (15.80)	40 (10.44)	0.01
Provide advice to patients on their health issue	464 (29.80)	89 (23.24)	0.01
Share information with patients	346 (22.22)	56 (14.62)	0.00
Other	235 (15.09)	118 (30.81)	0.00

Participants could select more than one option; * Includes all public facilities: primary healthcare clinics, community health centres, district hospitals, tertiary hospitals; ~ Includes all private health facilities: primary care clinics, medical practices, pharmacies, hospitals, alternative providers, NGOs, others; ^ For each cadre, the remaining participants indicated that they did not use their phone for this type of communication

Supplementary table 7: Purposes for which messaging apps are used to communicate with patients, by geographic place of work (n (%))

**Purpose**	**Rural**	**Urban**	**Peri-urban**	**chi-square p-value**
Share appointment times and reminders with patients	63(32.98) ~	742 (46.78)	62(37.80)	0.00
Contact patients who have defaulted on treatment	39(20.42)	252 (15.89)	27 (16.46)	0.28
Send test results to patients	42 (21.99)	215 (13.56)	29 (17.68)	0.00
Provide advice to patients on their health issue	57 (29.84)	437 (27.55)	59 (35.98)	0.07
Share information with patients	39 (20.42)	322 (20.30)	41 (25.00)	0.37
Other	30 (15.71)	301 (18.98)	22 (13.41)	0.14

[#] Participants could select more than one option; ~ For each place of work, the remaining participants indicated that they did not use their phone for this type of communication

Supplementary table 8: Healthcare workers' receipt of a mobile phone data allowance from their employer, by place of employment and location of health facility (n (%))

<table>
<tr><th rowspan="2"></th><th rowspan="2"></th><th colspan="2">Received an allowance</th><th rowspan="2">chi-square p-value</th></tr>
<tr><th>Yes</th><th>No</th></tr>
<tr><td rowspan="2">Place of employment</td><td>Private health facilities ¤</td><td>344 (22.71)</td><td>1171 (77.29)</td><td rowspan="2">0.00</td></tr>
<tr><td>Public health facilities*</td><td>47 (12.70)</td><td>323 (87.30)</td></tr>
<tr><td rowspan="3">Location of health facility</td><td>Rural</td><td>47 (26.26)</td><td>132 (73.74)</td><td rowspan="3">0.04</td></tr>
<tr><td>Urban</td><td>302 (19.62)</td><td>1237 (80.38)</td></tr>
<tr><td>Peri-urban</td><td>42 (25.00)</td><td>126 (75.00)</td></tr>
</table>

¤ Includes all private health facilities: primary care clinics, medical practices, pharmacies, hospitals, alternative providers, NGOs, others; * Includes all public facilities: primary healthcare clinics, community health centres, district hospitals, tertiary hospitals
